# Descriptive analysis of long-term survival after aortic valve replacement for dialysis patients: importance of renal pathologies and age

**DOI:** 10.1007/s11748-024-02011-3

**Published:** 2024-03-07

**Authors:** Kaoru Matsuura, Hiroyuki Yamamoto, Goro Matsumiya, Noboru Motomura

**Affiliations:** 1 Department of Cardiovascular Surgery, Chiba University Hospital, 1-8-1 Inohana, Chuo Ward, Chiba City, Chiba Prefecture 260-0856 Japan; 2 Department of Healthcare Quality Assessment, Graduate School of Medicine, The University of Tokyo, Tokyo, Japan; 3 Department of Cardiovascular Surgery, Toho University Sakura Medical Center, Chiba, Japan

**Keywords:** Dialysis, Aortic valve replacement, Descriptive analysis, Long-term prognosis

## Abstract

**Objectives:**

This study analyzed the long-term survival of dialysis patients undergoing AVR using the Japanese National Clinical Database with additional survival data.

**Methods:**

De-novo AVR for dialysis-dependent patients between 2010 and 2012 who were registered in the Japan Cardiovascular Surgery Database were included. Concomitant aortic surgery and transcatheter aortic valve replacement were excluded. An additional questionnaire was sent to each hospital regarding the underlying kidney disease, the duration of dialysis initiation to the surgery, and clinical outcomes. The Kaplan–Meier survival curve was descriptively shown for all cohorts and each renal pathology. Furthermore, we compared the incidence of bioprosthetic valve failure in patients who were < 65 years old (group Y) and ≧65 years old (group O).

**Results:**

Of these 1529 patients, diabetic nephropathy was 517, chronic glomerulonephritis was 437, and renal sclerosis was 210, regarding renal pathology. 1, 3, and 5-year survival in each pathology was 78.4%, 58.6%, 45.9% in diabetic nephritis, 78.8%, 68.4%, 58.2% in chronic glomerulonephritis, 79.0%, 67.8%, 52.1% in renal sclerosis, and 74.4%, 62.6%, 49.2% in others. Active infectious endocarditis was more prevalent in group Y (O 2.7% vs. Y 9.6%). The incidence of bioprosthetic valve failure requiring re-hospitalization was too small to analyze. 1, 3, and 5-year survival was 76.0%, 63.4%, 49.2% in group O and 74.3%, 64.2%, and 47.7% in group Y.

**Conclusions:**

Long-term survival of AVR for dialysis-dependent was higher in patients with chronic glomerulonephritis and lower in patients with diabetic nephritis than in other pathologies.

## Introduction

The number of patients in the United States requiring chronic hemodialysis (HD) for end-stage renal disease (ESRD) has been increasing [1–3]. The Japanese annual dialysis data report shows that the elderly population, in particular, requires HD [4]. Approximately one-quarter of dialysis patients die from heart failure, with aortic stenosis (AS) being the most common cardiovascular condition among HD patients [5]. Aortic valve replacement (AVR) has been the established treatment for AS for many years. While the operative mortality of AVR is higher for HD patients compared to non-HD individuals, the long-term survival after AVR for HD patients remains unknown [6].

ESRD can result from various causes, such as diabetic nephropathy, chronic glomerulonephritis, or renal sclerosis. Survival after AVR may differ among patients with these different renal pathologies, although this aspect has not been investigated yet. Additionally, the use of bioprosthetic valves has increased in the general population, coinciding with the emergence of transcatheter aortic valve replacement (TAVR). Moreover, there has been a rise in AS patients due to improved accessibility to valve therapy over the past few decades [7]. Therefore, it is crucial to understand the long-term survival after AVR for HD patients with different renal pathologies. Furthermore, it is important to investigate the long-term survival after AVR using bioprosthetic valves in both young and elderly HD patients.

In a previous study, we utilized a national database to compare the long-term survival of dialysis patients who underwent AVR with mechanical or biological valves [8]. The present study uses a national database in Japan to provide a descriptive analysis of the long-term outcomes of AVR for HD patients based on renal pathology and age.

## Methods

The study design, including the data registration project, was approved by the institutional review board of the Chiba University Graduate School of Medicine Ethical Committee on November 25th, 2020, with approval number 3334.

### Data collection

JCVSD (Japanese Cardiovascular Surgery Database) is a national database containing clinical datasets from nearly all cardiovascular units in Japan [1–3]. The data manager of each hospital was responsible for registering the clinical data to the central office. The data entry rate was monitored annually in the central office, and the accuracy of registered data was maintained through a data audit; administrative office members and investigators randomly visited a participating hospital. Informed consent was obtained from all patients at each participating hospital.

The patients who required chronic HD and underwent AVR between 2010 and 2012 were included in this study. Concomitant annular enlargement, coronary artery bypass grafting (CABG), and mitral surgery were included, and TAVR, redo surgery, and concomitant thoracic aortic surgery were excluded from this study.

An additional data processing system was constructed, and additional data were registered by each hospital online. Additional data consist of the pathophysiology of renal failure (diabetic nephropathy, glomerulonephritis, renal sclerosis, and others), the time between the initiation of dialysis and surgery (years), death, and prosthetic valve failure requiring admission. An online additional data registration system was opened between October 29th, 2019 and October 30th, 2020. The Data Utilization Committee of the JCVSD Organization approved the use of data for the present study.

### Statistical methods

All continuous values are expressed as mean ± SD or median (IQR) as appropriate. The different patient groups performed a comparative analysis of background patient data. Pearson's chi-squared, Kruskal–Wallis, and Fisher's exact tests were used. Moreover, the value of two-sided *p* < 0.05 was used to indicate significance.

This study consisted of two studies. The first study investigated the long-term survival after AVR for HD patients in each renal pathology. The second study was designed to investigate the bioprosthetic valve failure requiring re-admission and long-term survival after the AVR using the bioprosthetic valve for HD patients < 65 years old and >  = 65 years old.

## Results

The total number of hospitals that had target cases was 382 in Japan. Of these, 252 hospitals showed intention to participate in this study. Finally, 1596 patients performed in 216 hospitals were registered in this study. Data registration was completed in 90.6% of index patients. Therefore, a total of 1529 patients performed in 211 hospitals were analyzed in this study.

Of these 1529 patients, the number of patients whose renal pathology was diabetic nephropathy was 517, chronic glomerulonephritis was 437, renal sclerosis was 210, and others were 365. Baseline patient characteristics stratified by the different renal pathologies are shown in Table 1. The patients with renal sclerosis were older, and patients with chronic glomerulonephritis were younger (*p* < 0.001). The prevalence of diabetes mellitus treated medically was higher in diabetic nephropathy (*p* < 0.001). The prevalence of coronary artery disease was higher in diabetic nephropathy (40.2%) and less in chronic glomerulonephritis (25.4%) (*p* < 0.001). Hypertension was less prevalent in chronic glomerulonephritis (*p* < 0.001). Peripheral artery disease was more common in diabetic nephropathy (*p* = 0.001). Carotid artery disease and cerebral vascular disease were less common in chronic glomerulonephritis (*p* = 0.03, *p* = 0.002). The years after initiation of HD were longer in chronic glomerulonephritis (*p* < 0.001). Large body mass index was more prevalent in diabetic nephropathy. The mechanical valve was used more in chronic glomerulonephritis (*p* = 0.01).

**Table 1:** Patient characteristics (stratified by the renal pathology)

	Total	Diabetic nephropathy	Chronic glomerulonephritis	Renal sclerosis	Others	*p* value	Test
	*N* = 1529	*N* = 517	*N* = 437	*N* = 210	*N* = 365		
Age							
− 59	138 (9.0%)	49 (9.5%)	43 (9.8%)	10 (4.8%)	36 (9.9%)	< 0.001	Pearson's chi-squared
60–64	202 (13.2%)	65 (12.6%)	58 (13.3%)	15 (7.1%)	64 (17.5%)		
65–69	311 (20.3%)	106 (20.5%)	107 (24.5%)	32 (15.2%)	66 (18.1%)		
70–74	390 (25.5%)	131 (25.3%)	118 (27.0%)	46 (21.9%)	95 (26.0%)		
75–79	317 (20.7%)	113 (21.9%)	82 (18.8%)	63 (30.0%)	59 (16.2%)		
80–	171 (11.2%)	53 (10.3%)	29 (6.6%)	44 (21.0%)	45 (12.3%)		
Male	997 (65.2%)	358 (69.2%)	269 (61.6%)	140 (66.7%)	230 (63.0%)	0.064	Pearson's chi-squared
Diabetes medicated	360 (23.5%)	309 (59.8%)	27 (6.2%)	8 (3.8%)	16 (4.4%)	< 0.001	Fisher's exact
Coronary disease	478 (31.3%)	208 (40.2%)	111 (25.4%)	64 (30.5%)	95 (26.0%)	< 0.001	Pearson's chi-squared
Myocardial infarction	110 (7.2%)	52 (10.1%)	22 (5.0%)	16 (7.6%)	20 (5.5%)	0.011	Pearson's chi-squared
Coronary bypass	567 (37.1%)	251 (48.5%)	132 (30.2%)	74 (35.2%)	110 (30.1%)	< 0.001	Pearson's chi-squared
Active endocarditis	40 (2.6%)	15 (2.9%)	9 (2.1%)	2 (1.0%)	14 (3.8%)	0.17	Fisher's exact
Hypertension	1160 (75.9%)	434 (83.9%)	305 (69.8%)	169 (80.5%)	252 (69.0%)	< 0.001	Pearson's chi-squared
Peripheral artery disease	305 (19.9%)	132 (25.5%)	78 (17.8%)	38 (18.1%)	57 (15.6%)	0.001	Pearson's chi-squared
Carotid artery disease	81 (5.3%)	36 (7.0%)	18 (4.1%)	15 (7.1%)	12 (3.3%)	0.038	Pearson's chi-squared
Cerebrovascular disease	209 (13.7%)	92 (17.8%)	45 (10.3%)	33 (15.7%)	39 (10.7%)	0.002	Pearson's chi-squared
Low ejection fraction (< 30%)	111 (7.3%)	35 (6.8%)	41 (9.4%)	10 (4.8%)	25 (6.8%)	0.16	Pearson's chi-squared
NYHA (class3or4)	549 (35.9%)	195 (37.7%)	146 (33.4%)	79 (37.6%)	129 (35.3%)	0.52	Pearson's chi-squared
Chronic lung disease	240 (15.7%)	79 (15.3%)	65 (14.9%)	40 (19.0%)	56 (15.3%)	0.55	Pearson's chi-squared
Years after initiation of HD	8 (4–15)	6 (3–10)	13 (6–19)	6 (3–12)	9 (4–16)	< 0.001	Kruskal–Wallis
Mitral surgery	294 (19.2%)	88 (17.0%)	92 (21.1%)	49 (23.3%)	65 (17.8%)	0.15	Pearson's chi-squared
Shock	48 (3.1%)	21 (4.1%)	9 (2.1%)	10 (4.8%)	8 (2.2%)	0.11	Fisher's exact
Liver dysfunction	36 (2.4%)	5 (1.0%)	10 (2.3%)	5 (2.4%)	16 (4.4%)	0.012	Fisher's exact
Atrial fibrillation	220 (14.4%)	68 (13.2%)	65 (14.9%)	28 (13.3%)	59 (16.2%)	0.6	Pearson's chi-squared
Smoking	152 (9.9%)	60 (11.6%)	39 (8.9%)	16 (7.6%)	37 (10.1%)	0.34	Pearson's chi-squared
Rheumatic	44 (2.9%)	15 (2.9%)	18 (4.1%)	4 (1.9%)	7 (1.9%)	0.26	Fisher's exact
Body mass index							
− 18.5	329 (21.5%)	73 (14.1%)	104 (23.8%)	45 (21.4%)	107 (29.3%)	< 0.001	Pearson's chi-squared
18.5–25.9	1094 (71.6%)	386 (74.7%)	314 (71.9%)	153 (72.9%)	241 (66.0%)		
26–	106 (6.9%)	58 (11.2%)	19 (4.3%)	12 (5.7%)	17 (4.7%)		
Emergency							
Elective	1422 (93.0%)	472 (91.3%)	416 (95.2%)	194 (92.4%)	340 (93.2%)	0.26	Fisher's exact
Emergent	32 (2.1%)	15 (2.9%)	4 (0.9%)	6 (2.9%)	7 (1.9%)		
Urgent	75 (4.9%)	30 (5.8%)	17 (3.9%)	10 (4.8%)	18 (4.9%)		
Aortic stenosis	1338 (87.5%)	470 (90.9%)	376 (86.0%)	183 (87.1%)	309 (84.7%)	0.028	Pearson's chi-squared
Mechanical valve	692 (45.3%)	222 (42.9%)	224 (51.3%)	84 (40.0%)	162 (44.4%)	0.019	Pearson's chi-squared

**Fig. 1:**
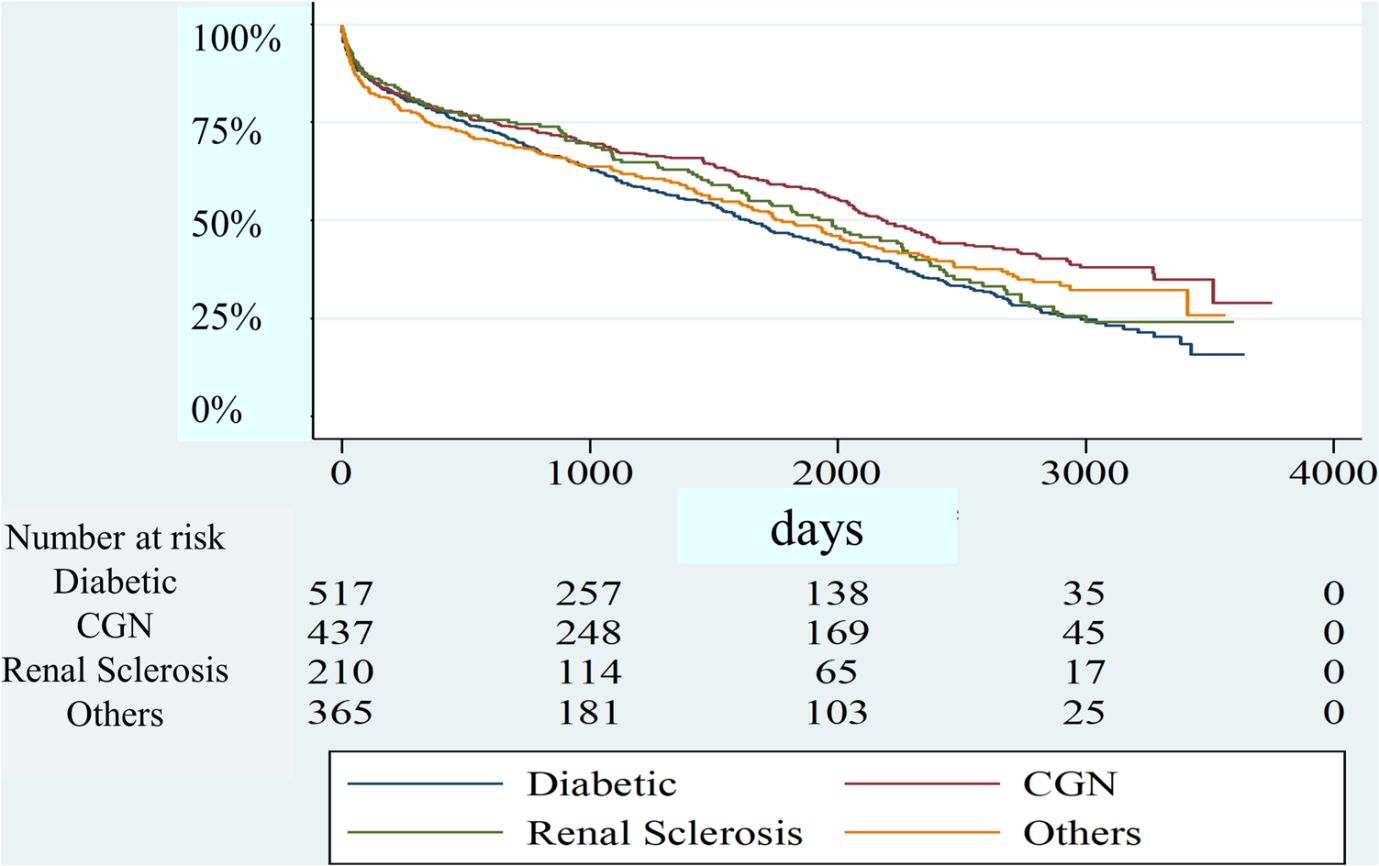
Survival curve stratified by the renal pathology

A Kaplan–Maier survival estimates curve stratified by the different renal pathologies is shown in Fig. 1. Median survival time was 4.50 years in diabetic nephropathy, 5.97 years in chronic glomerulonephritis, 5.41 years in renal sclerosis, and 4.86 years in others. 1, 3, 5-year survival rate was 78.4%, 58.6%, 45.9% in diabetic nephropathy, 78.8%, 68.4%, 58.2% in chronic glomerulonephritis, 79.0%, 67.8%, 52.1% in renal sclerosis, and 74.4%, 62.6%, 49.2% in others.

Eight-hundred and thirty-seven patients underwent AVR with a bioprosthetic valve, and 692 underwent AVR with a mechanical valve. These 837 patients were analyzed in the second study. Of these patients, 92 patients were < 65 years old at the time of operation (group Y), and 745 patients were >  = 65 years old (group O). Baseline patient characteristics stratified by the different renal pathologies are shown in Table 2. Coronary artery disease was more prevalent in the older population (*p* = 0.05). Active infectious endocarditis was more prevalent in the younger population (*p* = 0.002).

**Table 2:** Patient characteristics (stratified by the age of 65 years old)

	Total	< 65yo	> = 65yo	*p* value	Test
	*N* = 837	*N* = 92	*N* = 745		
Male	558 (66.7%)	61 (66.3%)	497 (66.7%)	0.94	Pearson's chi-squared
Diabetes medicated	203 (24.3%)	24 (26.1%)	179 (24.0%)	0.70	Fisher's exact
Coronary disease	254 (30.3%)	20 (21.7%)	234 (31.4%)	0.057	Pearson's chi-squared
Myocardial infarction	59 (7.0%)	3 (3.3%)	56 (7.5%)	0.19	Fisher's exact
coronary bypass	297 (35.5%)	24 (26.1%)	273 (36.6%)	0.046	Pearson's chi-squared
Active endocarditis	29 (3.5%)	9 (9.8%)	20 (2.7%)	0.002	Fisher's exact
hypertension	627 (74.9%)	65 (70.7%)	562 (75.4%)	0.32	Pearson's chi-squared
Peripheral artery disease	167 (20.0%)	23 (25.0%)	144 (19.3%)	0.20	Pearson's chi-squared
Carotid artery disease	51 (6.1%)	7 (7.6%)	44 (5.9%)	0.49	Fisher's exact
Cerebrovascular disease	128 (15.3%)	16 (17.4%)	112 (15.0%)	0.55	Pearson's chi-squared
Low ejection fraction (< 30%)	60 (7.2%)	9 (9.8%)	51 (6.8%)	0.29	Fisher's exact
NYHA (class3 or 4)	305 (36.4%)	35 (38.0%)	270 (36.2%)	0.73	Pearson's chi-squared
Chronic lung disease	145 (17.3%)	11 (12.0%)	134 (18.0%)	0.15	Pearson's chi-squared
Renal pathology					
Diabetic nephropathy	295 (35.2%)	32 (34.8%)	263 (35.3%)	0.011	Fisher's exact
Chronic glomerulonephritis	213 (25.4%)	21 (22.8%)	192 (25.8%)		
Renal sclerosis	126 (15.1%)	6 (6.5%)	120 (16.1%)		
Others	203 (24.3%)	33 (35.9%)	170 (22.8%)		
Years after initiation of HD	7 (3–13)	10 (5–17.5)	7 (3–13)	0.001	Wilcoxon rank-sum
Mitral surgery	139 (16.6%)	20 (21.7%)	119 (16.0%)	0.16	Pearson's chi-squared
Shock	29 (3.5%)	6 (6.5%)	23 (3.1%)	0.12	Fisher's exact
Liver dysfunction	24 (2.9%)	5 (5.4%)	19 (2.6%)	0.17	Fisher's exact
Atrial fibrillation	126 (15.1%)	10 (10.9%)	116 (15.6%)	0.23	Pearson's chi-squared
Smoking	69 (8.2%)	17 (18.5%)	52 (7.0%)	< 0.001	Pearson's chi-squared
Rheumatic	17 (2.0%)	0 (0.0%)	17 (2.3%)	0.24	Fisher's exact
Body mass index					
− 18.5	175 (20.9%)	17 (18.5%)	158 (21.2%)	0.20	Fisher's exact
18.5–25.9	614 (73.4%)	66 (71.7%)	548 (73.6%)		
26-	48 (5.7%)	9 (9.8%)	39 (5.2%)		
Emergency					
Elective	770 (92.0%)	81 (88.0%)	689 (92.5%)	0.25	Fisher's exact
Emergent	18 (2.2%)	3 (3.3%)	15 (2.0%)		
Urgent	49 (5.9%)	8 (8.7%)	41 (5.5%)		
Aortic stenosis	728 (87.0%)	71 (77.2%)	657 (88.2%)	0.003	Pearson's chi-squared

**Fig. 2:**
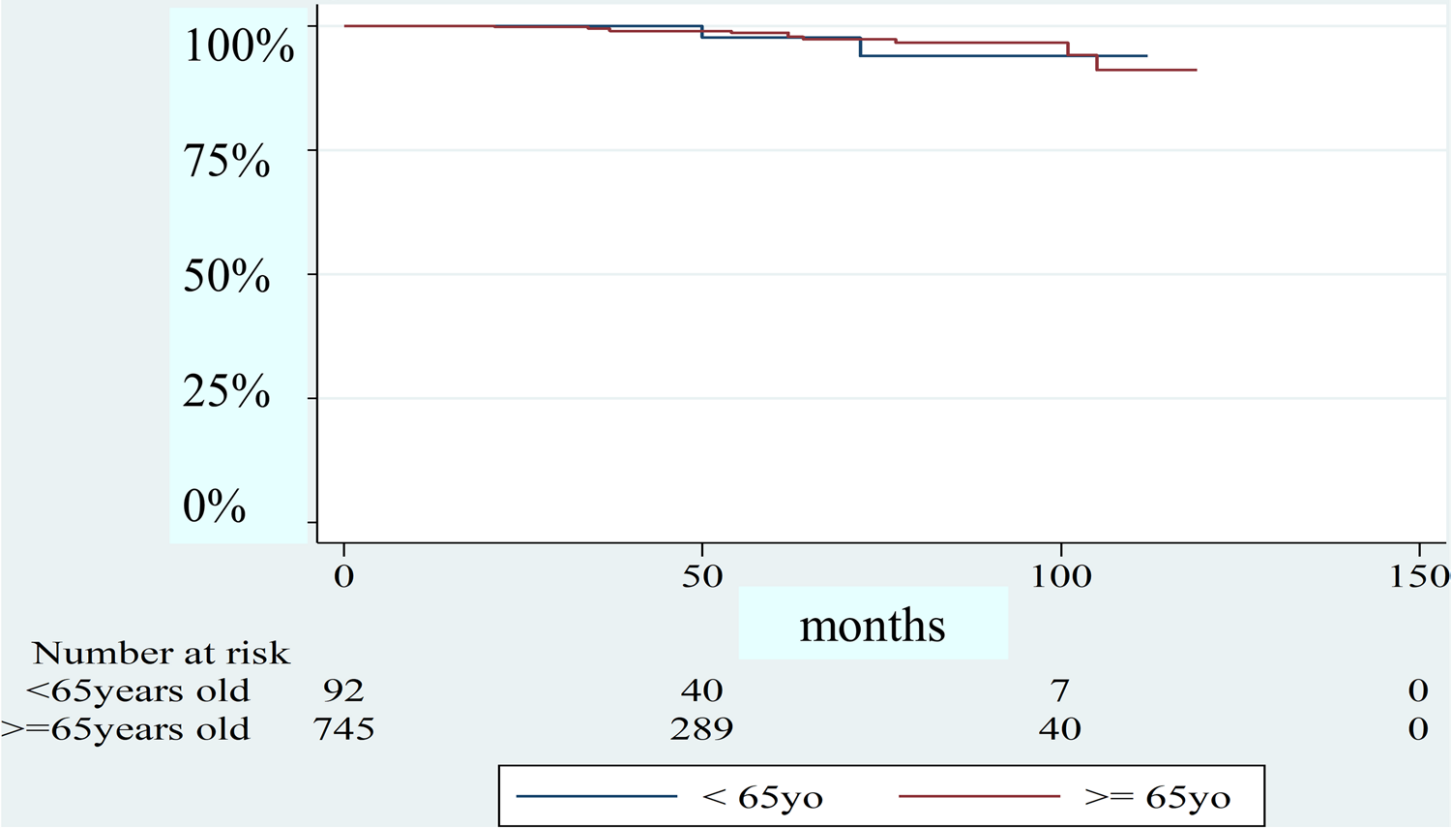
Freedom from the bioprosthetic valve failure requiring re-hospitalization

**Fig. 3:**
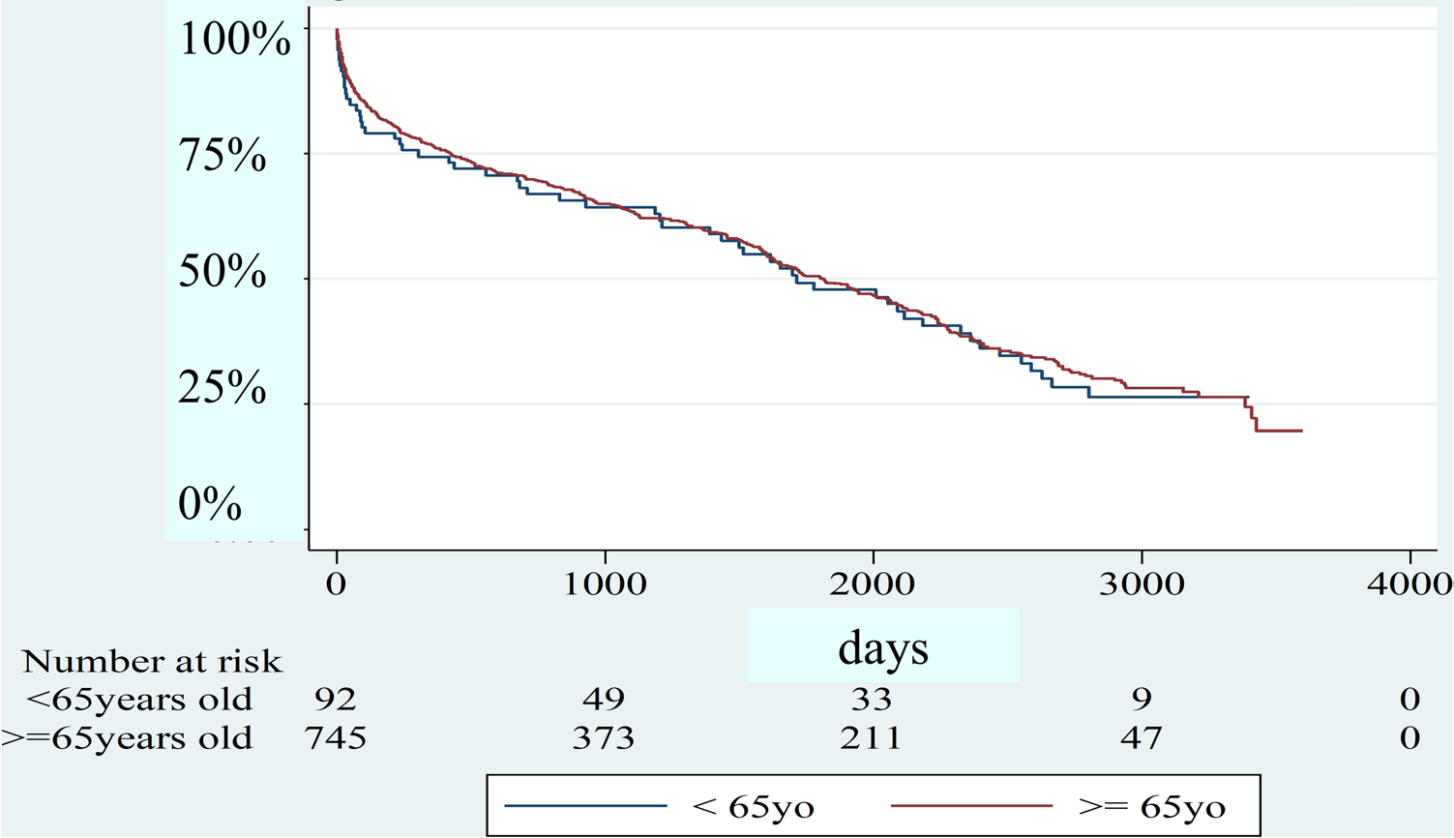
Survival curve stratified by age < or >  = 65 years old

Regarding renal pathology, renal sclerosis was more common in group O (*p* = 0.01). The years after initiation of HD were longer in group Y (*p* = 0.001). More patients in group Y had a smoking habit (*p* = 0.001). Moreover, aortic stenosis was more prevalent in group O (*p* = 0.003). Figure 2 shows the event-free curve of bioprosthetic valve failure requiring re-admission. It was difficult to analyze because the number of this event was exceedingly small. Figure 3 shows the Kaplan–Meyer survival curve stratified by the age of sixty-five. The median survival time was 4.69 years in group Y and 4.96 years in group O. Furthermore, the 1, 3, 5-year survival rate was 74.3%, 64.2%, and 47.7% in group Y and 76.0%, 63.4%, and 49.2% in group O.

## Discussion

In terms of the cause of death among HD patients, a quarter of them died of heart failure. Furthermore, it is undeniable that aortic valve stenosis may be related to unknown and sudden deaths [4]. The average life expectancy of the general population in Japan is 15.8 years for men and 20.8 years for women at 70, but this is 6.2 years for men and 7.1 years for women on HD [4].　The same statistics for the US show that life expectancy is 13–14 years for the general population at age 70 but 3–4 years for dialysis patients, indicating that the life expectancy of dialysis patients is relatively long in Japan[5, 6].

We have previously reported the long-term survival after AVR with mechanical or bioprosthetic valves in dialysis patients using the same cohort as this study [7]. Long-term survival of dialysis-dependent patients was similar regardless of whether mechanical or bioprosthetic valves were implanted. The incidences of cerebral and gastrointestinal bleeding were twofold higher in the mechanical valve group. This present article focuses on details, which were pathophysiology and age. Japanese guidelines recommend bioprosthetic valves as Class IIa for patients over 65. Moreover, it did not clearly recommend the valve choice for patients 60–65. However, bioprosthetic valves are also used in younger patients, and comparing the results would provide beneficial clinical information. For this reason, we divided the bioprosthetic valve use cohort into those over 65 years of age and those under 65 years of age. The use of bioprosthetic valves was more common in IE patients, which may be because of the concern of bleeding complications by anticoagulation, which is necessary for mechanical valves.

The distribution of the pathophysiology of renal disease in Japan was almost the same as in the present study [4]. Until now, no reports have analyzed the prognosis of dialysis patients after aortic valve replacement by primary kidney disease. In the present report, patients with diabetic nephropathy had the worst prognosis, and those with chronic glomerulonephritis had the best prognosis, with a difference of about 1.5 years. The difference in prognosis length is especially essential when considering treatment options for HD patients.

The treatment of AS patients is increasing as TAVR has become safer and more commonly available. In Japan, TAVR for dialysis patients was covered by insurance in 2021. The availability of TAVR for dialysis patients has had two significant impacts on their treatment choices. The first is the availability of TAVR as an initial treatment for AS in HD patients. Second, valve-in-valve TAVR for bioprosthetic valve failure is now available as an option.

Regarding the pros and cons of therapeutic intervention for AS in HD patients, a group at Kyoto University reported an extensive study of the natural history of patients with AS under the name of the CURRENT AS Study [8]. According to this study, the 5-year survival rate for HD patients with AS was 44% and 70% for non-HD patients. However, the 5-year mortality rate was 60.6% for patients who underwent AVR and 75.5% for patients who received medical treatment alone, suggesting that AVR can improve prognosis [8]. On the other hand, short-term outcomes of AVR in HD patients have been reported to be poorer than in non-HD patients [9]. A report from the US indicated that in-hospital mortality for AVR was 6.2% in non-HD patients and 11.8% in HD patients [10]. However, the number of AVR procedures in HD patients is increasing yearly, and surgical mortality rates are also decreasing in the US [10].

On the other hand, the Japanese National Database also reports that the number of AVR for HD patients increased from 2369 in 2013–2014 to 2834 in 2015–2016 [11]. The surgical mortality rate decreased from 11.7% in 2013–2014 to 10.7% in 2015–2016 [11]. Looking at the choice of valve type for AVR in HD patients in Japan, bioprosthetic valves increased from 64.9% in 2013–2014 to 69.3% in 2015–2016 [11]. Despite the increasing trend, the use of bioprosthetic valves in the general population was 78.7% in 2015–2016 [11]. By comparison, bioprosthetic valves tended to be less common in HD patients. This is likely a result of concerns about early bioprosthetic valve deterioration in HD patients.

On the other hand, Szerlip et al. reported that the observed-to-expected mortality ratio of the TAVR procedure was lower in dialysis patients than in non-dialysis patients [12]. The surgical results of TAVR in HD patients have been reported previously [12–17]. Most reports have reported high operative mortality rates for TAVR in HD patients, as Kobrin et al. reported in their propensity-matched study of HD and non-HD patients, that mortality was higher in HD patients [13]. The reason for the higher risk of TAVR in HD patients is that HD patients have more calcified lesions in the access route, which may result in more alternative access. In a Japanese multi-center report, Maeda et al. found that 10% of HD patients underwent TAVR with alternative access [14, 15]. However, the latest report on TAVR in HD patients found a dramatic decrease in operative mortality [18]. Ogami et al. reported that the 30-day mortality rate for TAVR in HD patients decreased from 11.1% in 2012 to 2.5% in 2016 [18]. On the other hand, this report also shows a low 5-year survival rate of 20% [18].

This present study provides important information in selecting treatment for AVR in dialysis patients. Diabetic nephropathy has a shorter prognosis than chronic glomerulonephritis by about 1.5 years, and the other is that the prognosis does not seem to differ much with age. In addition, now that the valve-in-valve TAVR is available, the choice of the initial treatment should be made after careful consideration of whether a second treatment is feasible. In other words, if valve-in-valve TAVR is feasible, the bioprosthetic valve AVR could be the therapeutic option for the selected younger patients.

After bioprosthetic valve replacement in HD patients, the most crucial concern is early bioprosthetic valve failure [19]. We adopted the definition of bioprosthetic valve failure as bioprosthetic valve failure requiring hospitalization. This is because some cases of bioprosthetic valve failure may be inoperable. Although the number of events of bioprosthetic valve failure requiring hospitalization was low in this study, bioprosthetic valve failure may be among the causes of unexplained deaths and sudden deaths. However, even if the likelihood of bioprosthetic valve failure is high, the long-term prognosis for AVR in HD patients is not long, as shown in this study. So, initial bioprosthetic valve use is always worth considering, including the possibility of the valve-in-valve TAVR. In such a case, the renal pathology in the present study should be considered. In such cases, the prognosis in this study according to the underlying disease may be beneficial. When performing AVR in HD patients, it is necessary to predict the longevity of the bioprosthetic valve as well as the life expectancy of the patient.

### Limitations

The present study was descriptively constructed from the beginning and is not statistically comparable by etiology or age. Therefore, the results cannot be used to make definitive clinical recommendations. Lack of information on the detailed cause of death is also a limitation. We did not ask for a detailed cause of death for two reasons. One reason is that we wanted to keep it simple to increase the data entry rate. The other reason is that we expected that the causes of death in dialysis patients, in general, include sudden death as well as infection and heart failure and that the detailed cause of death would often be unknown in the first place. Another limitation is the definition of bioprosthetic valve failure. We could not include the echocardiographic data to reduce the burden of additional data processing. The endpoint definitions for aortic valve clinical research were updated [20]. Bioprosthetic valve dysfunction is defined in detail because it is clinically critical. Future research on bioprosthetic valve dysfunction in dialysis patients is essential.

## Conclusions

Long-term survival of AVR for dialysis-dependent was higher in patients with chronic glomerulonephritis and lower in patients with diabetic nephritis than in other pathologies.

## Data Availability

The data that support the findings of this study are available from the corresponding author, KM, upon reasonable request.

## Abbreviations

AS: Aortic stenosis
HD: Hemodialysis
AVR: Aortic valve replacement
SVD: Structural valve deterioration
TAVR: Transcatheter aortic valve replacement
JCVSD: Japan Cardiovascular Surgery Database
CABG: Coronary artery bypass grafting
NYHA: New York Heart Association
SD: Standard deviation
IQR: Inter-quartile range
